# Amyloid beta pathology induces astrocytic pTDP-43 mislocalization and disrupts TDP-43-regulated cryptic exon transcripts

**DOI:** 10.3389/fnagi.2026.1766448

**Published:** 2026-04-15

**Authors:** Zeinab Rafiee, Jessica Santiago, Emelie Andersson, Oskar Hansson, Malin Wennström

**Affiliations:** 1 Cognitive Disorder Research Unit, Department of Clinical Sciences Malmö, Lund University, Malmö, Sweden; 2 Clinical Memory Research Unit, Department of Clinical Sciences Malmö, Lund University, Lund, Sweden

**Keywords:** Alzheimer’s disease, amyloid-beta, cryptic exon splicing, proteinopathy, pTDP-43

## Abstract

**Background:**

While amyloid-β (Aβ) and tau are hallmark pathologies of Alzheimer’s disease (AD), TDP-43 proteinopathy is increasingly recognized as an important contributor, occurring in up to 57% of AD cases and associated with accelerated cognitive decline. TDP-43 regulates RNA splicing, and its mislocalization leads to cryptic exon inclusion and loss of canonical protein function. While neuronal TDP-43 pathology has been well studied, its role in astrocytes remains less understood. Recent findings suggest increased phosphorylated TDP-43 (pTDP-43) inclusions in astrocytic endfeet in AD and a bidirectional interaction between Aβ and TDP-43, promoting mutual aggregation.

**Methods:**

We analyzed pTDP-43 immunoreactivity (IR) in astrocytic perivascular end-feet, nuclei, and cytosol in hippocampal sections from 3-month-old and 18-month-old App^NL–F/NL–F^ mice and 18-month-old wild-type controls using ImageJ. *In vitro*, primary fetal human astrocytes were exposed to oligomeric Aβ42, and changes in cytosolic and nuclear pTDP-43 IR were quantified via ImageJ, while TDP-43 and pTDP-43 protein levels were measured using an in-house ELISA. Expression of canonical transcripts ATG4B and KALRN, involved in autophagy and synaptic support, was assessed by qPCR. Corresponding protein-level changes were evaluated using in-house ELISA.

**Results:**

Our findings demonstrate significantly higher pTDP-43 accumulations in astrocytic nuclei, cytosol, and endfeet in 18-month-old App^NL–F/NL–F^ mice compared to age-matched wild-type mice. Astrocytes exposed to oligomeric Aβ42 showed elevated cytosolic pTDP-43 IR and total pTDP-43 protein levels. Concurrently, expression of canonical ATG4B and KALRN transcripts was significantly reduced, which was accompanied by corresponding decreases in protein levels.

**Conclusion:**

Our findings demonstrate that pTDP-43 accumulates in astrocytic nuclei, cytosol, and endfeet in the presence of AD pathology. The observed Aβ-induced increase in cytosolic pTDP-43 and transcript disruption suggests a mechanistic link contributing to autophagy impairment and cytoskeletal changes in astrocytes, potentially exacerbating AD progression.

## Introduction

Alzheimer’s disease (AD) is the leading cause of dementia worldwide, accounting for 60–70% of all cases, and its prevalence continues to rise with global aging populations (Meneses et al., 2021). Neuropathologically, AD is defined by extracellular amyloid-β (Aβ) plaques and intracellular neurofibrillary tangles (NFT) composed of hyperphosphorylated tau (p-tau) (Hardy and Selkoe, 2002; Janelidze et al., 2016; Ross and Poirier, 2004). Aβ is produced from a transmembrane Aβ precursor protein (APP) through sequential cleavage by β- and γ-secretases (Janelidze et al., 2016; Meneses et al., 2021). Among the various Aβ isoforms, Aβ42 is the most prone to aggregation and predominates in neuritic plaques, which are closely linked to neuronal loss and cognitive decline (Pinho et al., 2014). While fibrillar forms of Aβ42 constitute the core of these plaques, numerous studies have identified its soluble oligomeric forms as the most neurotoxic species (Tolar et al., 2021). Its accumulation in the AD brain results from overproduction of Aβ and/or impaired clearance, either through glial uptake (Ries and Sastre, 2016) or dysfunction of the blood–brain barrier (BBB) and glymphatic system (Iliff et al., 2012; Rasmussen et al., 2018). While Aβ and phosphorylated tau (p-tau) are central to AD pathology, growing evidence highlights the contribution of additional proteinopathies to disease heterogeneity and severity. Notably, inclusions of TAR DNA-binding protein 43 (TDP-43) have emerged as a critical pathological feature, present in approximately 30–57% of AD cases and associated with accelerated cognitive decline and hippocampal atrophy (Arai et al., 2006; Josephs et al., 2014; Meneses et al., 2021). The pTDP-43 inclusions in AD are found predominantly in the CA1 region (Rafiee et al., 2025; Santiago et al., 2025; Zhou et al., 2025), a hippocampal subfield highly vulnerable to neurodegeneration, where damage alone can cause severe memory impairment and dementia (Leverenz et al., 2002).

TDP-43 is a 43 kDa heterogeneous nuclear ribonuclear protein (hnRNP) composed of 414 amino acids and is encoded by the TARDBP gene located on chromosome 1 (1p36.22) (Meneses et al., 2021). Much like other hnRNPs, TDP-43 regulates gene expression and various aspects of RNA processing, including RNA splicing, mRNA turnover, RNA trafficking, and microRNA (miRNA) biogenesis (Buratti and Baralle, 2008; Chew et al., 2019; Fukushima et al., 2019; Higashi et al., 2013). TDP-43 targets over 4,000 different mRNA transcripts (Sephton et al., 2011), ranging from disease-associated transcripts (Gu et al., 2017) to its own mRNA transcript (Ayala et al., 2011). One of its key nuclear functions is to repress the incorporation of non-conserved cryptic exons during pre-mRNA splicing (Polymenidou et al., 2011; Seddighi et al., 2024). Under stress or disease conditions, this function is lost as nuclear TDP-43 undergoes phosphorylation (pTDP-43), truncation, and mislocalization to the cytoplasm, where it aggregates into insoluble inclusions (Buratti, 2018). The loss of nuclear TDP-43 function inevitably leads to the inappropriate inclusion of non-conserved exons, resulting in the production of faulty or unstable proteins and a reduction in canonical protein levels (Prudencio et al., 2020). The best-established examples of affected transcripts are Stathmin-2 (STMN2) and UNC13A, which are essential for neuronal axonal maintenance and synaptic vesicle release, respectively (Agra Almeida Quadros et al., 2024). Inclusion of cryptic exons in these transcripts is strongly correlated with pTDP-43 burden in AD and frontotemporal lobar degeneration (FTLD), but not with Aβ or tau deposition, highlighting a distinct mechanism of pathogenesis (Agra Almeida Quadros et al., 2024). Building on these findings, recent studies suggest that cryptic exon inclusion may serve as a sensitive and specific biomarker for TDP-43 pathology. This is particularly relevant for neurodegenerative diseases such as amyotrophic lateral sclerosis (ALS), frontotemporal lobar degeneration (FTLD) (Arai et al., 2010), and limbic-predominant age-related TDP-43 encephalopathy neuropathologic changes (LATE-NC) (Nelson et al., 2023), where TDP-43 aggregation is a defining pathological hallmark, but also in the large subset of AD cases.

Although TDP-43 inclusions are primarily found in neurons, an increasing number of studies show that astrocytes are also affected (Arai et al., 2010; Licht-Murava et al., 2023; Nelson et al., 2023) and postmortem analyses have revealed pTDP-43 inclusions in astrocytic endfeet of AD patients (Santiago et al., 2025). The functional impact of these inclusions remains to be fully understood, but a study on transgenic AD mice models has shown a link between TDP-43 accumulation in astrocytic TDP-43 and progressive memory loss (Licht-Murava et al., 2023). Given the ubiquitous role of TDP-43 in RNA splicing, it is likely that astrocytic transcripts also can be cryptically spliced, causing a reduction in important canonical proteins. However, most reports on TDP-43 related cryptic splicing described changes in transcripts related to neuronal events, while cryptic exon products from glial cells remain largely unexplored. Nevertheless, single-cell RNA sequencing data from the Human Protein Atlas indicate that some of the reported transcripts can also be found in astrocytes. Examples of such transcripts are *ATG4B* (Autophagy Related 4B Cysteine Peptidase) and *KALRN* (Kalirin, serine/threonine-protein kinase with Dbl and pleckstrin homology domain), which are involved in autophagy (Torres et al., 2024) and synaptic dysfunction or cognitive impairment (Remmers et al., 2014; Russell et al., 2018), respectively.

But the accumulation of pTDP-43 inclusions in astrocytes can also have other functional consequences. We have recently shown that the presence of perivascular Aquaporin 4 (AQP4) in AD patients is reduced along with increased density of pTDP-43 inclusions in astrocytic endfeet. Given the crucial role of AQP4 in the clearance of Aβ by the glymphatic system, we proposed that pTDP-43 inclusions in the astrocytic endfeet can play a role in Aβ accumulation (Santiago et al., 2025). Indeed, emerging evidence points to a bidirectional and pathogenic relationship between Aβ and TDP-43. Experimental studies show that soluble Aβ can facilitate the oligomerization of TDP-43, which in turn cross-seeds Aβ into toxic complexes (Shih et al., 2020). *In vivo* studies show, on the other hand, that lentiviral expression of Aβ in the rat motor cortex induces TDP-43 pathology and co-localization between intracellular Aβ and TDP-43 (Herman et al., 2011). In our own recent study, we report significant correlations between Aβ42 and pTDP-43 levels in patients with stable mild cognitive impairment and patients with vascular dementia (Rafiee et al., 2025). Hence, it seems like there is a complex relationship between Aβ and TDP-43, but the mechanism behind this relationship, whether this relationship is direct or indirect, and/or affects astrocytic processes, is not known.

Based on the crucial role for astrocyte in Aβ clearance, the previous finding of pTDP-43 inclusion in astrocytes, the suggested relationship between TDP-43 and Aβ aggregation, and the potential loss of TDP-43 function on astrocytic RNA splicing, we hypothesize that Aβ42 pathology, directly or indirectly, induces astrocytic pTDP-43 pathology, causing a reduction in levels of cryptic exon-bearing proteins. To test this hypothesis, we examined perivascular pTDP-43 as well as astrocytic nuclear and cytosolic pTDP-43 inclusions in the hippocampus of App^NL–F/NL–F^ knock-in mice before and after the formation of Aβ plaques and analyzed the cellular localization of pTDP-43 as well as the expression of canonical ATG4B and KALRN transcripts in cultured human astrocytes exposed to low concentrations of oligomer Aβ42.

## Materials and methods

### Animal model

The App ^NL–F/NL–F^ knock-in mice express humanized Aβ with the Swedish (APP K670N/M671L) and Iberian (APP I716F) mutations, under the endogenous mouse amyloid precursor protein (APP) promoter. In this experiment, the App^NL–F/NL–F^ knock-in mice and wild type (WT) littermates were housed in groups of 2–5 mice per cage under a 12:12-h light/dark cycle with food and water provided *ad libitum*. The App^ NL–*F/NL*–*F*^ knock-in mice at 3 months (*n* = 5, whereof *n* = 3 female and *n* = 2 male), 18 months (*n* = 6, whereof *n* = 2 female and *n* = 4 male) of age and WT littermates at 18 months of age (*n* = 6, whereof *n* = 3 female and *n* = 3 male) were euthanized under deep anesthesia (medetomidine, 1 mg/kg; ketamine, 120 mg/kg; i.p.) following transcardial perfusion with ice-cold 0.1 M phosphate buffer thereafter brains were collected and post-fixed in 4% paraformaldehyde and cryoprotected in 30% sucrose, then cut as free-floating sagittal sections (30 μm) from midline on a cryostat. Experiments approved by Malmö/Lund Committee for Animal Experiment Ethics (Dnr 8798-202).

### Immunostaining

Sections were washed with phosphate-buffered saline containing potassium (KPBS), followed by incubation in blocking solution, consisting of 5% normal goat serum (Jackson Immunoresearch), Triton X-100 0.25% (Fisher Scientific) in KPBS (KPBS+) for 1 h at room temperature. Then, the tissue was incubated with primary antibodies, chicken anti-GFAP (Millipore,1:1,000), rabbit anti-pTDP-43 (1:400, Proteintech), and blocking solution overnight at 4°C. Afterward, the tissue was washed with KPBS+ and incubated for 2 h with the appropriate fluorochrome-conjugated secondary antibody at room temperature. Biotinylated goat anti-chicken antibody (VectorLaboratories) was used as a secondary antibody in the GFAP immunostaining, followed by incubation with Streptavidin 488 (VectorLaboratories) for 1 h. And finally, after washing, the section was mounted with Vectashield Set mounting medium containing 4’,6-diamidino-2-phenylindole (DAPI) (VectorLaboratories) for nuclear staining.

### ImageJ area fraction analysis

To assess the area fraction of pTDP−43 associated to GFAP-positive endfeet enwrapping vessels and pTDP−43 within astrocytic nuclei and cytosol, single plane images (five images for perivascular pTDP-43 and ten images for astrocytic nuclei and cytosol) with three color channels were captured from the molecular layer (ML) of cornu ammonis (CA1) of three section (2.5-3-2 lateral to the midline) using a 40 × objective in an Olympus AX70 light microscope equipped with an Olympus DP72 camera. All images were analyzed using the Fiji software (ImageJ2 version 2.9.0). The pTDP−43 quantification was performed as previously described (Santiago et al., 2025). In short, five GFAP-enwrapped small vessels per mouse (outer diameter between 10 and 15μm) were randomly selected. The continuous GFAP-positive perivascular sleeve bordering the lumen was manually traced using the Fiji polygon tool, nuclei were excluded, and a fixed threshold was applied to quantify the pTDP-43 load in the cytoplasm. For astrocytic nuclei and cytosolic analysis, five randomly selected GFAP-positive cells per mouse were outlined using the polygon tool to define astrocyte boundaries, and regions of interest (ROIs) were added to the ROI manager. Nuclear areas were defined using DAPI staining and added to the ROI manager as well. pTDP-43 signal inside the DAPI-positive ROIs was measured for nuclear localization. Cytosolic pTDP-43 was identified by subtracting nuclear ROIs from the corresponding GFAP-positive ROIs. A uniform threshold was applied across all images to detect pTDP-43 inclusions. For each ROI, the area fraction of pTDP-43 (percentage of the region occupied by pTDP-43 signal) per mice were calculated using the “Measure” function in Fiji.

### In vitro

#### Cell culture and stimulation

Hippocampal astrocytes (HA) of fetal human origin (ScienCell, Carlsbad, CA, #1830) were grown at a density of 30,000 cells/mL in a 0.5% poly-L-lysine prepared cell culture 8-well chambers and 48-well plate (Thermoscientific, Nunclon *™* Delta Surface) in astrocyte medium (ScienCell, #0413) with 2% fetal bovine serum (FBS), 10% astrocyte growth supplement and 10% penicillin/streptomycin solution. The medium was replaced with fresh medium the next day and then every second day until cells were confluent (90% or more). Human HA cells (ScienCell, Carlsbad, CA, #1830 were stimulated with oligomeric Aβ42 (Synthetic peptide, 1μM, Alexotech #686). To prepare the peptides for the stimulation, oligomeric Aβ42 was dissolved in NaOH (20 mM, pH 12) and ddH_2_O. Phosphate buffer (200 mM, pH 7) and NaCl (5 M) were then added. ddH_2_O was subsequently added to yield a final peptide concentration of 100 μM peptide solution was vortexed for 20 min at RT to form Oligomers. The vehicle was used as control for oligomeric Aβ42 stimulation was prepared according to the peptide preparation without the peptides: NaOH (20 mM, pH 12), ddH_2_O, phosphate buffer (200 mM, pH 7), and NaCl (5 M) (Hays et al., 2016). The medium of cultured astrocytes in chambers and plates was replaced with medium without FBS and incubated for 2 h at 37°C to serum starve the cells. Cells were then stimulated with 1 μM Aβ42 and vehicle for 19 h at 37°C, as this dose (Hou et al., 2011) and duration (Byman et al., 2019; Nielsen et al., 2010) have been shown to be less toxic and do not significantly increase necrosis within the experimental time frame.

#### Lactate dehydrogenase cytotoxicity assay

Lactate dehydrogenase cytotoxicity assay (LDH) activity was measured to investigate astrocytic cell death after stimulation with oligomeric Aβ42 and vehicle. The LDH assay (Roche, #82081400) was performed according to the manufacturer’s instructions. Following stimulation, the medium of the stimulated cell culture was collected and placed in a 96-well plate (three independent experiments, *n* = 5 wells per condition). LDH reaction mixture was added to each well and incubated at Room temperature (RT) on agitation. LDH activity was measured at OD 450 using a microplate reader. Data was gathered using Gen5 (version 2.05).

#### Immunocytochemistry

Stimulated cells in 8-well chambers were rinsed once in phosphate-buffered saline with calcium and magnesium (PBS ++), followed by fixation (2% paraformaldehyde, 15 min, RT). Cells were permeabilized with blocking buffer (0.1% of saponin in 1%BSA/PBS, 15 min), washed, and blocked with blocking buffer (30 min). Afterward, incubated with primary antibody against pTDP-43 (dilution 1:500; Proteintech), chicken anti-GFAP (dilution 1:1,000; Proteintech), in blocking buffer for 2 h. Cells were rinsed in PBS++ and then incubated with corresponding secondary antib odies for 1 h at RT: goat anti-rabbit (dilution 1:200; Invitrogen), biotin-conjugated goat anti-chicken (dilution 1:500; Vector). Cells were then washed in PBS and incubated with Streptavidin (1:500; Vector) for 1 h, RT, and finally, mounted with mounting medium with DAPI (VECTORSHIELD, #ZK1003) for nuclear staining. cells were incubated (overnight, +4°C) in primary antibody: rabbit anti TDP-43 rabbit anti AQP4 (dilution 1:1,000; Sigma-Aldrich).

#### ImageJ analysis of the cells

To assess the area fraction of pTDP−43 inside astrocytic nuclei and cytosol, 10 images of each experiment were randomly captured in the green channels, whereafter images were also captured in the red channel using a 40 × objective in an Olympus AX70 light microscope equipped with an Olympus DP72 camera. All images were analyzed using the Fiji software (ImageJ2 version 2.9.0). For astrocyte identification, GFAP staining was used to define astrocytic boundaries. GFAP-positive cells were manually outlined with the polygon tool, and the ROIs were added to the ROI manager. To define nuclei, DAPI staining was thresholded to select nuclear regions and then added to the ROI manager. Nuclear pTDP-43 was identified by applying the DAPI ROIs to the pTDP-43 channel and excluding what was outside. Cytosolic pTDP-43 was identified by subtracting the nuclear ROI from the GFAP ROI, thereby isolating the cytoplasmic area. To quantify the inclusions, a consistent global threshold was applied across all images to detect pTDP-43 inclusions. For each ROI, the area fraction (% of area occupied by pTDP-43 signal) was measured using the “Measure” function in Fiji.

### Protein measurement in lysed cells

The protein concentrations of pTDP43 in the lysed cell samples were measured using an In-house-made enzyme-linked immunosorbent assay (ELISA). Stimulated cells were lysed using the Mammalian Cell Lysis Kit (MCLB) kit (Sigma Aldrich, #0000272502) according to the kit instructions. The samples, in duplicate, were diluted 1:20 in TBS (50 mM Tris-HCl, 150 mM NaCl, pH 8) and incubated at 4°C overnight in optically clear 96-well flat-bottom microplates (Nunc, ThermoScientific, #442404). The peptide used to generate the assay antibody was used as a standard diluted 200–12.5 pg/mL (22309-1-BP, Proteintech). The following day, the plates were blocked for 1 h at RT with blocking solution (BSA) (1% bovine serum albumin in TBS), followed by incubation with rabbit polyclonal p-TDP-43 Ser409/410 (Proteintech, United States) diluted 1:1,000 in blocking buffer for 2 h at RT with agitation. Finally, goat-anti-rabbit horseradish peroxidase secondary antibody (Agilent Technologies, #41584785) diluted 1:2,000 in blocking buffer was added to the plate and incubated for 1 h at RT with agitation. The plates were then developed using TMB microwell peroxidase (SeraCare KPL, #5120-0048); the reaction was stopped with 1 M H2SO4, and the optical density was measured at 450 nm and 540 nm using the spectrophotometer Eon™ (Biotek). Levels of TDP-43, KALRN, and ATG4B were semi-quantitatively analyzed using an in-house ELISA, where the cell samples were analyzed using the above protocol after diluting the samples (1:20, 1:40, and 1:20, respectively) and using the appropriate primary antibody (TDP-43 Proteintech, #10782-2-AP, KALRN Proteintech, #19740-1-AP) and ATG4B Proteintech, #15131-1-AP.

### Gene expression analysis

The Aβ42 oligomer -stimulated HA cells grown in 6-well plates (three individual experiments in two replicates) were lysed on ice using Qiazol lysis reagent (Qiagen, Venlo, the Netherlands) and transferred to microcentrifuge tubes. The total RNA from the lysed cells was purified using RNeasy Plus Universal Mini Kit (Qiagen, Venlo, the Netherlands), according to the manufacturer’s instructions. RNA Purity and concentration were quantified using Take 3™ and Eon™ (Biotek, Winooski, VT), and the concentration was adjusted with RNase-free water. Preparation of cDNA was performed using the Maxima first-strand cDNA synthesis kit (Life Tech, Carlsbad, CA) according to the manufacturer’s instructions and thereafter mixed with Maxima probe/ROX QPCR mastermix (Life Tech, Carlsbad, CA). A total of 8 ng/μL of cDNA was amplified by real-time PCR. Kalirin, RhoGEF kinase (*KALRN*) (Hs01118227_mL, exon 8–9 junction), autophagy related 4B cysteine peptidase (*ATG4B*) (Hs00367088_mL, exon 7–8 junction) and housekeeping genes including ribosomal protein L13A (*RPL13A*) (HS04194366_g1) and Glyceraldehyde-3-phosphate dehydrogenase hydroxymethylbilane synthase (*GAPDH*) *(*Hs02758991_g1*)* (Applied Biosystems, Foster City, CA) were used. The RT-qPCR reactions were carried out using Viia™ 7 system (Applied Biosystems, Foster City, CA) and the relative expression in mRNA level was calculated using the 2^–ΔCt^ method (Schmittgen and Livak, 2008) and normalized against the geometric mean of RPL13A and GAPDH as references gene.

### Statistical analysis

The Prism software (version 10, GraphPad) was used for statistical analysis and graphical representation. The Kolmogorov–Smirnov test was performed to assess normal distribution. For normally distributed data (*in vivo* data), a one-way ANOVA followed by the Tukey test was used. For non-normally distributed data (*in vitro* data), the Mann–Whitney U test was performed. Normally distributed data are represented as means ± standard deviations, while non-normally distributed data are represented as medians. Individual values are shown in both cases. Data are represented as scatter plots. A value of *p* < 0.05 was considered significant.

## Results

### pTDP-43 inclusions increase after Aβ plaque formation in App^NL–F/NL–F^ mice

**FIGURE 1:**
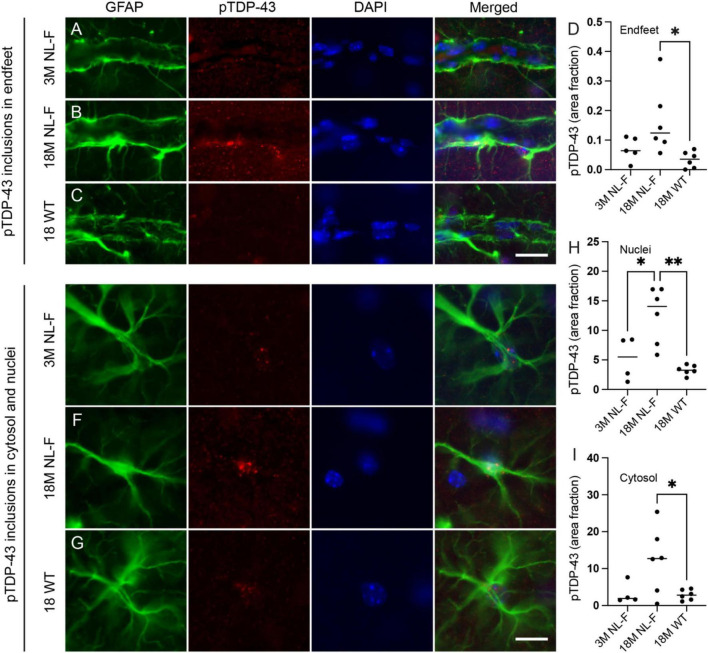
Perivascular and cellular pTDP-43 in the hippocampus of App^NL–F/NL–F^ knock-in mice. Representative co-immunofluorescence images of the CA1 region of the hippocampus in 3-month-old App^NL–F/NL–F^ (3M NL-F) **(A,E)**, 18-month-old App^NL–F/NL–F^ (18M NL-F) **(B,F)**, and 18-month-old wild-type (18M WT) **(C,G)** mice show staining for GFAP (green), pTDP-43 (red), and DAPI (blue). pTDP-43–positive inclusions associated with GFAP-positive astrocytic endfeet **(A–C)**, GFAP-positive astrocyte processes and astrocyte nuclei **(E–G)**. Quantitative analysis of perivascular pTDP-43 area fraction across groups is shown **(D)**, comparing (*n* = 5) 3M NL-F, (*n* = 6) 18M NL-F, and (*n* = 6) 18M WT mice, and cytosolic **(H)** and nuclei **(I)** pTDP-43 area fraction comparing (*n* = 4) 3M NL-F, (*n* = 6) 18M NL-F, and (*n* = 6) 18M WT mice. Data was analyzed using one-way ANOVA with Tukey post-test, and each dot represents the mean value of pTDP-43 area fraction in each animal. *p < 0.05, **p < 0.01, Scale bars 20 μm.

To investigate if perivascular pTDP-43 accumulation is associated with Aβ plaque formation, we analyzed the hippocampal CA1 region of App^NL–F/NL–F^ knock-in mice at two time points: one preceding initial Aβ plaque formation and cognitive impairment, and one well after plaques have developed and cognitive impairment is detectable. Since Aβ deposition in this model begins around 6 months of age and cognitive deficits are detectable at 18 months of age (Saito et al., 2014), we chose to analyze brain sections from 3-month-old to 18-month-old App^NL–F/NL–F^ mice. To ensure that pTDP-43 accumulation was not simply a consequence of aging, we also analyzed 18-month-old wild-type mice. The immunofluorescence staining of the CA1 region revealed perivascular pTDP-43 inclusions associated with GFAP staining in all subgroups (Figures 1A–C). Quantification of the perivascular pTDP-43 area fraction further showed that 18-month-old App^NL–F/NL–F^ mice exhibited higher signal intensity of pTDP-43 GFAP-positive endfeet compared to age-matched wild-type controls and a trend to higher signal intensity compared to 3-month-old App^NL–F/NL–F^ mice (*p* = 0.1261) (Figure 1D). We also analyzed the pTDP-43 area fractions in the cytosol and nucleus of GFAP-positive astrocytes in the same area (Figures 1E–G). The cytosolic pTDP-43 area fractions were significantly elevated in 18-month-old App^NL–F/NL–F^ mice compared to both 3-month-old App^NL–F/NL–F^ and 18-month-old WT animals, while nuclear pTDP-43 area fractions in 18-month-old App^NL–F/NL–F^ mice were only significantly higher compared to age-matched wild-type controls and only a trend to higher area fractions compared to 3-month-old App^NL–F/NL–F^ (*p* = 0.0803) (Figures 1H,I).

### Cellular localization and levels of astrocytic pTDP-43 are altered after Aβ42 exposure

**FIGURE 2:**
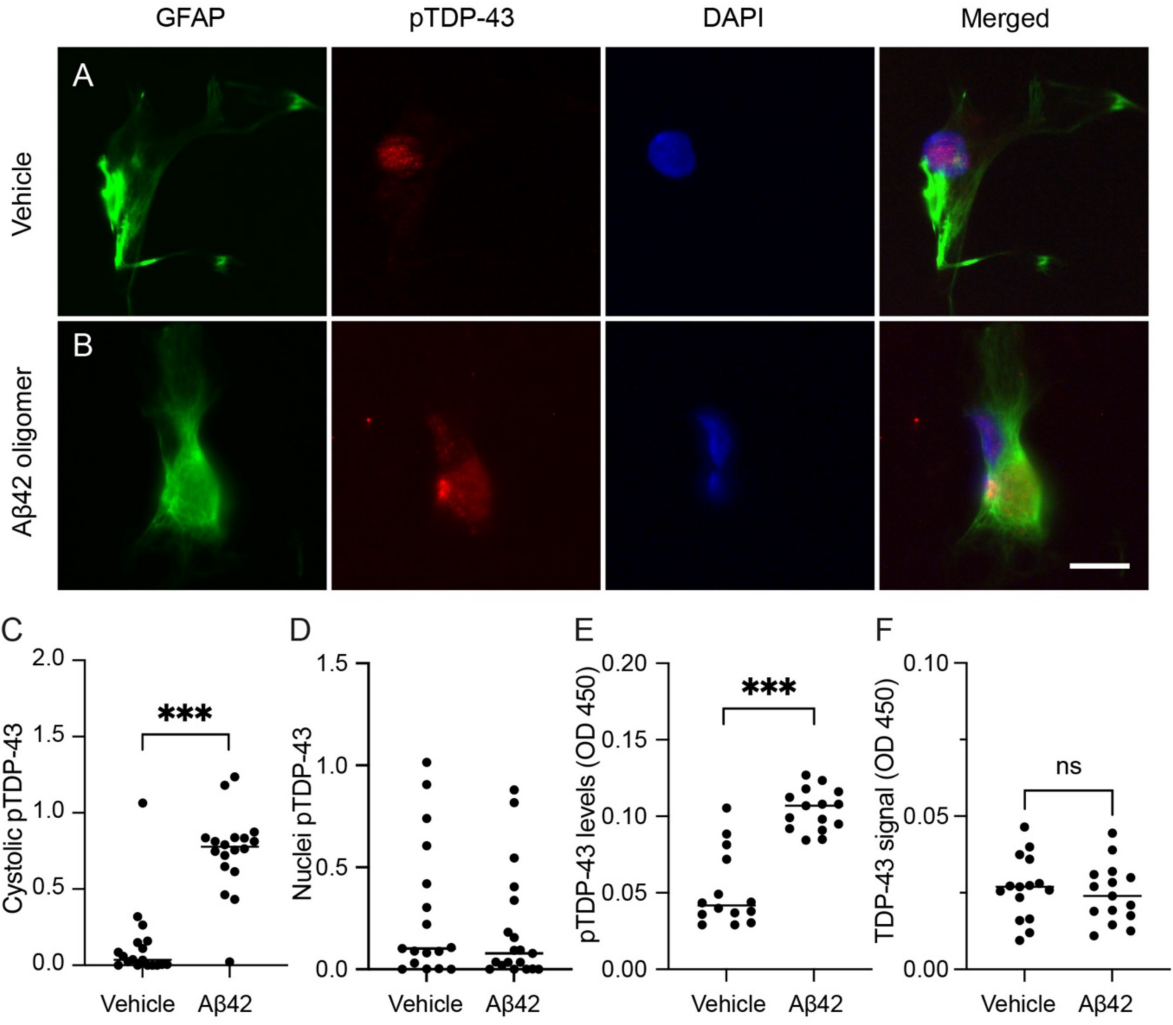
Phosphorylated TDP-43 (pTDP-43) expression in human fetal hippocampal astrocytes (HA) after oligomer Aβ42 exposure. Representative immunofluorescence images of HA cells co-stained with pTDP-43 (red), GFAP (green), and DAPI (blue) following treatment with vehicle **(A)** or oligomer Aβ42 **(B)**. **(C)** Graph demonstrating the area fraction of pTDP-43 immunoreactivity in the cytosol of GFAP-positive cells. **(D)** Graph demonstrating the area fraction of pTDP-43 immunoreactivity in the nuclei of GFAP-positive cells. **(E)** Measurement of pTDP-43 protein levels in cell lysates using an in-house enzyme-linked immunosorbent assay (ELISA). **(F)** Measurement of TDP-43 protein signal in cell lysates using an in-house ELISA. Experiment in **(C,D)** was performed twice, and each dot represents pTDP-43 in GFAP-expressing astrocyte (*n* = 10 images per experiment). The experiment in E and F was performed 3 times, with 5 independent replicates in each experiment. Each dot represents one independent replicate. Data were analyzed using the Mann-Whitney test. ***p < 0.001. Scale bar: 10 μm.

Next, we investigated the direct effect of aggregated and toxic Aβ42 on astrocytic pTDP-43. Cultured HA cells stimulated with oligomeric Aβ42 were immunostained against pTDP-43 together with the astrocytic marker GFAP and the nuclear marker DAPI. In vehicle-treated cells, pTDP-43 was faintly detected (Figure 2A), whereas Aβ42-treated cells displayed a strong cytoplasmic pTDP-43 signal in GFAP-positive astrocytes (Figure 2B). Because TDP-43 function and toxicity depend on its subcellular distribution, we quantified cytosolic and nuclear pTDP-43 separately. The analysis revealed a markedly higher intensity of cytosolic pTDP-43 in Aβ42-treated astrocytes compared with vehicle controls (Figure 2C), while no change in Aβ42 stimulation was observed in the proportion of pTDP-43-positive nuclei (Figure 2D). Since pTDP-43 pathology in neurodegeneration is often associated not only with mislocalization but also with altered abundance, we next measured pTDP-43 levels in lysates of stimulated astrocytes. Using an in-house ELISA, we found that oligomeric Aβ42-treated astrocytes revealed higher amounts of pTDP-43 compared with vehicle-treated cells (Figure 2E). We also used a semi quantitative, in house–developed ELISA to measure total TDP 43, to determine whether the observed changes are specifically related to alterations in phosphorylation. No alterations in TDP 43 signal were observed when comparing lysates from oligomeric Aβ42 treated cells with those from vehicle treated controls (Figure 2F). Finally, to ensure that these effects were not secondary to cell death, we assessed membrane integrity using a lactate dehydrogenase (LDH) release assay. No difference in LDH release was detected between groups, suggesting that the observed changes in pTDP-43 localization and release occurred independently of extensive cell death.

### Oligomeric Aβ42 reduces expression of canonical KALRN and ATG4B

**FIGURE 3:**
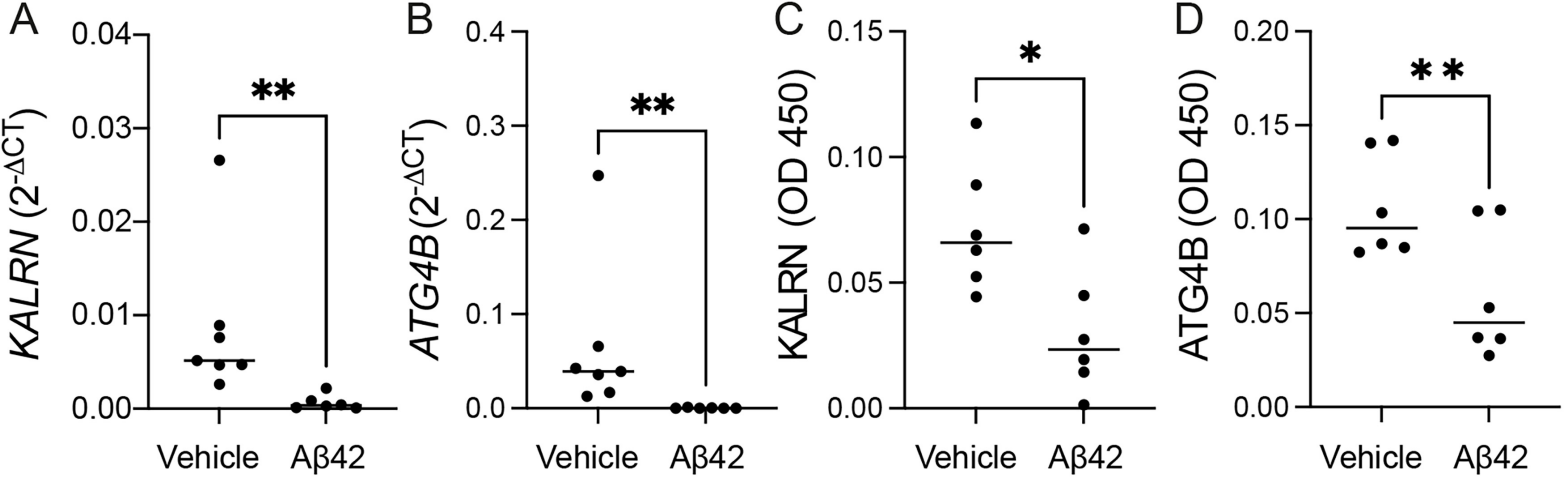
Analysis of ATG4B and KALRN expression in HA cells after Aβ42 exposure. Relative mRNA levels of KALRN **(A)** and ATG4B **(B)** were quantified by qPCR using the 2^–ΔCT^ method. The protein level of KALRN **(C)** and ATG4B **(D)** was measured by an in-house enzyme linked immunosorbent assay (ELISA). The experiment was performed 3 times, with 2 independent replicates. Statistical significance was determined by Mann-Whitney test. *p < 0.05, **p < 0.01.

To examine the potential impact of oligomer Aβ42 on TDP-43-related cryptic exons-bearing transcripts in astrocytes, we quantified canonical *KALRN* (Figure 3A) and *ATG4B* (Figure 3B) mRNA in HA cells exposed to oligomeric Aβ42. Indeed, both transcripts were significantly reduced in astrocytes exposed to Aβ42 compared to vehicle control. Finally, to determine whether this transcriptional effect translates into protein expression, we investigated the protein level of KALRN (Figure 3C) and ATG4B (Figure 3D) in the same samples. Levels of both ATG4B and KALRN reduced significantly.

## Discussion

In the current study, we combined *in vivo* analysis, using a transgenic AD mouse model, with *in vitro* experiments on human fetal primary astrocytes to investigate the potential link between amyloid beta (Aβ) pathology and astrocytic pTDP-43 mislocalization and dysfunction. Our *in vivo* data showed that 18-month-old, but not 3-month-old, App^NL–F/NL–F^ mice exhibited significantly more perivascular GFAP-associated, cytosolic, and nuclear pTDP-43 inclusions compared to age-matched wild-type controls. This finding aligns with previous reports of increased neuronal and overall TDP-43 pathology in transgenic AD mouse models such as 3xTg (Herman et al., 2011) and 5xFAD (Gao et al., 2020), and suggests that Aβ pathology may contribute to TDP-43 aggregation not only in neurons but also in other brain cell types, including astrocytes.

The presence of pTDP-43 inclusions in astrocytic endfeet is consistent with our previous observations in AD patient tissue (Santiago et al., 2025). However, the current study does not clarify whether these inclusions arise from increased expression or mislocalization of intracellular astrocytic TDP-43, or whether other mechanisms are involved. Of note, there is currently no evidence in the literature for increased total TDP-43 levels in Alzheimer’s disease. Instead, proteomic (Hondius et al., 2016) and neuropathological studies consistently indicate that TDP-43 pathology in AD is characterized by mislocalization, aggregation, and hyperphosphorylation rather than altered protein abundance, suggesting that the observed increases in pTDP-43 are primarily due to enhanced phosphorylation rather than elevated expression (Gao et al., 2025). Interestingly, studies of individuals with limbic-predominant age-related TDP-43 encephalopathy (LATE) have shown that Lin bodies (TDP-43-positive micro-lesions) are frequently surrounded by reactive astrocytes, suggesting that astroglia may respond to and sequester TDP-43 aggregates near blood vessels (Shahidehpour et al., 2024). Moreover, astrocytes have been shown to take up and accumulate extracellular or “leaked” TDP-43 in disease (Lee et al., 2020; Prater et al., 2022). The astrocytic pTDP-43 inclusions observed at perivascular endfeet may therefore also result from active scavenging of extracellular aggregates or impaired trafficking of misfolded TDP-43, ultimately leading to inclusion formation.

To further explore whether astrocytic pTDP-43 accumulation in the presence of Aβ pathology can occur independently of extracellular uptake, we conducted *in vitro* studies using cultured astrocytes derived from human fetal hippocampus. Exposure to oligomeric Aβ42 led to a significant increase in cytosolic pTDP-43 levels, but no change in the overall TDP-43 levels, a finding that corresponds well with our *in vivo* result and further suggests a direct effect of toxic Aβ42 on TDP-43 phosphorylation within astrocytes.

Previous studies suggest that a mislocation of TDP-43 from the nuclei to the cytosol contributes to a loss of function (De Boer et al., 2021). In the current study, we found that Aβ pathology led to increased nuclei pTDP-43 *in vivo* and unaltered levels *in vitro*. These findings may contradict the loss of function idea, but although phosphorylation of TDP-43 is required for normal function (Eck et al., 2021; Keating et al., 2023), hyperphosphorylation and sequestration into nuclear inclusions led to insolubility and immobility of free functional nuclear TDP-43 (Keating et al., 2023). In addition, even subtle nuclear TDP-43 alterations have been linked to cryptic exon splicing in key genes in AD patients (Trautwig et al., 2025). Consistent with this, we found that oligomeric Aβ42 exposure significantly reduced mRNA levels of ATG4B and KALRN, which are two known targets of TDP-43 regulation. This reduction may reflect a loss of TDP-43 function, leading to the inclusion of cryptic exons, nonsense-mediated mRNA decay, and diminished canonical transcript levels (Torres et al., 2024; Trautwig et al., 2025). Our findings of protein levels of both ATG4B and KALRN are in line with this idea and further support the hypothesis that Aβ42 toxicity can initiate TDP-43-related RNA misprocessing in astrocytes, even in the absence of extensive nuclear clearance. Although additional work will be required to determine the functional consequences of these misprocesses, it is particularly interesting that we observed a reduction in ATG4B transcripts and protein levels. ATG4B is a key regulator of autophagy (Maruyama and Noda, 2018), a process essential for degrading damaged organelles and abnormal protein aggregates (Boya et al., 2013). Specifically, ATG4B is involved in autophagosome maturation and recycling, key steps in maintaining functional autophagy. This is particularly relevant given that impaired autophagy is a hallmark of AD pathology, leading to the accumulation of autophagic vacuoles and protein aggregates due to ineffective clearance (Kiraly et al., 2011; Torres et al., 2024). Notably, TDP-43 dysfunction has been shown to induce aberrant inclusion of a cryptic exon within the ATG4B transcript, resulting in impaired autophagy and promoting neurodegeneration (Torres et al., 2018). Consistent with this mechanism, elevated levels of the ATG4B cryptic exon correlate with advanced Braak stages in AD (Torres et al., 2020), highlighting the relevance of TDP-43–dependent cryptic splicing to AD pathobiology. Although cryptic exon inclusion in ATG4B has not yet been reported, the reduction in canonical ATG4B transcripts observed here may help explain the direct effects of Aβ42 on astrocytic autophagy reported by others (Kim et al., 2024). Such disruption could potentially create a vicious cycle, impairing Aβ clearance and accelerating proteinopathy.

KALRN, the other transcript found to be reduced in Aβ42-exposed astrocytes, encodes Kalirin, a protein best known for its role in dendritic spine formation, cytoskeletal dynamics, and synaptic plasticity (Colomer et al., 1997). Kalirin mRNA and protein levels are reported to be downregulated in the hippocampus of AD patients compared to controls (Calliari et al., 2024), which may be due to cryptic splicing. Recent studies showing truncated KALRN mRNA and reduced Kalirin protein in TDP-43 proteinopathies and AD cases support this hypothesis (Trautwig et al., 2025). Although Kalirin’s role in astrocytes remains unclear, its involvement in cytoskeletal regulation suggests that it may influence astrocyte morphology and behavior. In this case, Aβ42-induced reduction of Kalirin, as seen in our experimental study, could contribute to changes in astrocyte reactivity or motility.

### Limitations

While our findings offer valuable insights, they must be interpreted considering certain limitations. First, neither *in vitro* nor *in vivo* models can fully reproduce the complexity of human disease, but they remain essential tools for isolating and analyzing specific biological events that are otherwise impossible to disentangle in the human brain. Secondly, TDP-43 can be phosphorylated at up to 27 distinct sites (Kellett et al., 2025), yet our study focused exclusively on the S409/410 site. This site is, however, the most extensively characterized phosphorylation site in ALS, FTLD, and AD (Ko et al., 2024; Neumann et al., 2006; Nonaka et al., 2016). Therefore, the Aβ42-induced increase in pTDP-43 at S409/410 observed in both our *in vitro* and *in vivo* experiments likely reflect engagement of post-translational pathways similar to those implicated in human TDP-43 proteinopathies. Nonetheless, we cannot exclude the possibility that other phosphorylation sites may also contribute to astrocytic TDP-43 dysfunction and warrant further investigation. We further acknowledge that our study does not provide definitive evidence that the observed reduction in KALRN and ATG4B expression is due to cryptic splicing. Therefore, we encourage future studies employing exon-specific primers or long-read sequencing to conclusively determine whether Aβ-induced TDP-43 pathology drives specific cryptic splicing events in astrocytes. Finally, although our study shows molecular disturbances associated with Aβ42-induced pTDP-43 pathology, future studies will be necessary to determine how these changes translate into altered astrocyte function *in vivo* and in human tissue.

## Conclusion

This study identifies astrocytes as active contributors to TDP-43 pathology in the context of Alzheimer’s disease. The increased presence of astrocytic pTDP-43 inclusions in perivascular endfeet, cytosol, and nuclei, along with changes in TDP-43–regulated cryptic exon-bearing transcripts in response to Aβ42 exposure, suggests that Aβ42 induces glial dysfunction that may exacerbate disease progression. Furthermore, our *in vitro* data demonstrate that Aβ42 alone is sufficient to trigger TDP-43 phosphorylation and mislocalization in astrocytes, supporting a direct mechanistic link between Aβ toxicity and TDP-43 pathology. As TDP-43 dysfunction is increasingly recognized across neurodegenerative disorders, clarifying its role in AD remains essential. Therapeutic strategies targeting TDP-43-related mechanisms in astrocytes, in addition to neurons, may be necessary to effectively address AD cases with co-pathology and mitigate the accelerated neurodegenerative trajectory observed in these patients.

## Data Availability

The original contributions presented in the study are included in the article/supplementary material, further inquiries can be directed to the corresponding author.
